# Leveraging AI and data science to mitigate the respiratory health impacts of climate change in Africa: Organisation, costs, and challenges

**DOI:** 10.7189/jogh.14.03051

**Published:** 2024-12-06

**Authors:** Akinyimika O Sowunmi, Okechukwu Ignatius Eze, Uyi Osadolor, Alexander Iseolorunkanmi, Davies Adeloye

**Affiliations:** 1 School of Life and Health Sciences, Teesside University, Middlesbrough, UK; 2 International Business School, Teesside University, Middlesbrough, UK; 3 Department of Economics and Development Studies, Covenant University, Ota, Nigeria; 4 Covenant University Health Centre, Covenant University, Ota, Nigeria; 5 School of Life and Health Sciences, Teesside University, Middlesbrough, UK

The impacts of climate change on health, particularly respiratory health, have continued to generate concerns globally [1,2]. The United Nations Intergovernmental Panel on Climate Change, in its Fifth Assessment Report, identified climate change as a significant factor that amplifies existing health vulnerabilities [3]. It highlighted that human health is particularly sensitive to climate fluctuations, with recent observations showing that certain diseases are spreading into previously unaffected regions. In Africa, where healthcare systems already face substantial health burden, climate change exacerbates existing respiratory health issues like asthma and chronic obstructive pulmonary disease (COPD), with worsening symptoms being often reported [4,5].

In the context of public health, artificial intelligence (AI) such as machine learning (ML) models can predict the onset of respiratory conditions based on environmental data. It can also provide accessible, cost-effective solutions for health education, disease management, and healthcare delivery [6,7]. These tools have been deployed for self-management, facilitating remote health services, including automated diagnosis, and emotional support for mental health in Africa, where respiratory diseases such as asthma and COPD are prevalent [8]. Although ethical challenges such as data privacy have raised some concern, the ability to monitor and predict health risks exacerbated by climate change by analysing weather patterns, environmental factors, and health data real-time has been overall helpful for global health systems [7,8]. The unique potential of AI in bridging language barriers to improve health service delivery has also been widely reported [9], with substantial return on investment, as these technologies can help improve health and living standards through disease predictions and notifications, prompting relevant responses [6,7].

Despite the potential to mitigate climate risks and improve health outcomes, many African countries are lagging in the adoption and/or integration of AI and data science for strategic health development, including those related to respiratory medicine. Here we explore key issues around climate change, respiratory health, AI and data science in Africa, with a focus on the need for strategic planning, costing, and investment to fully leverage these technologies for sustainable health improvements on the continent.

## CLIMATE CHANGE, AI, AND DATA SCIENCE IN AFRICA’S PUBLIC HEALTH SPACE

AI and data science as data platforms or software solutions can simulate human-like interactions and process vast amounts of data through advanced natural language processing and ML [10]. They have recently begun to play transformative roles in public health, including for challenges related to climate change [11]. For example, ChatGPT, the AI application and chatbot developed by OpenAI, has been deployed in different disciplines, including health, and is now an integral part of several research and interventions [12].

Historically, AI has been applied across various public health domains, making its application to climate-related health issues a natural progression. Despite the seeming sparsity of health data and challenges with quality and representation in many African countries, AI and data science appear to be well suited to address Africa’s public health challenges if deployed meaningfully, potentially offering scalable, data-driven solutions to assess key population health risks, even in regions with limited infrastructure [8,12]. Leveraging advancements in ML, neural networks, and data analytics has resulted in the ability of AI to analyse vast data sets rapidly and predict disease trends, offering representative models for regions with sparse, unreliable data [13]. These benefits could be readily leveraged to address climate change and health impacts in Africa, particularly monitoring indoor and outdoor air quality, precipitation patterns, and rising temperatures [14]. For example, AI has been successfully used to monitor respiratory health issues during seasonal dust storms (e.g. harmattan in the Sahel part of Africa), which exacerbates asthma and allergies and the spread of respiratory symptoms [15]. Experts have reported that deploying relevant tools in AI and data science with an appropriate understanding of contextual factors, including health governance, finance, workforce, and policies on digital health strategies, holds transformative potential for improving many African countries’ capacity to address climate-related health risks [7,8]. However, the adoption of AI in this context across many African settings remains limited. An understanding of issues around the organisation and governance, the associated costs and contextual challenges, particularly on climate and health decisions, may be helpful for African healthcare systems.

## ORGANISATION, INFRASTRUCTURE, AND GOVERNANCE

Climate change poses significant challenges to communities and the environment in the Global South, with serious implications for respiratory health [16]. While international action and coordination are crucial, much can be done at regional and national levels to prepare for, mitigate, and/or address the impacts. Before developing country-level frameworks, however, government structures need to understand what mitigation and adaptation interventions are most feasible, effective and scalable. In terms of respiratory health, this requires comprehensive organisation, leadership and collaboration across key government sectors to prioritise respiratory health within the broader public health and climate change agenda [8]. These frameworks could also include clear regulatory standards for AI applications, ensuring proper data governance, privacy protection, and the ethical use of AI technologies [7]. Consequently, governments can ensure that AI is deployed responsibly and effectively to reduce health risks associated with climate change [8].

Kenya offers practical lessons from the deployment of AI-powered air quality monitoring systems in Nairobi [17]. This model, established through a public-private partnership involving the Kenyan government, local universities, and international firms, tracks pollution levels real-time and provides predictive insights into associated health risks. In coordination with environmental agencies, local health authorities were tasked with managing and interpreting the data, allowing for timely public health interventions. Governance of the system involves clearly defined roles for data collection, analysis, and response, with strong regulatory oversight to ensure data privacy and ethical use. The transparency of the project, alongside continuous community engagement, also helped build trust in the system, ensuring that data-driven interventions aligned with public health goals and environmental protection policies. Such initiatives could push AI to the forefront of government strategies across African countries, offering governments proactive solutions to mitigate health risks posed by climate change.

At the local level, communities are particularly vulnerable to the impacts of climate change, making it essential to build their capacity to adapt. Health initiatives in rural areas that use AI to collect and analyse health data provide another important example of how technology can transform healthcare delivery in underserved regions. One such initiative is the use of mobile health (mHealth) platforms, which leverage AI to gather real-time health data from rural populations. For example, Bradway and colleagues [18] developed a framework where local healthcare workers are trained to use mobile devices equipped with AI-powered diagnostic tools to track symptoms, manage chronic conditions, and identify potential health risks. This data are then uploaded to centralised databases where AI algorithms analyse it to detect patterns, predict outbreaks, and suggest targeted interventions.

Building local capacity by implementing education and training programmes focused on AI literacy and specialised training for healthcare professionals could be considered. For example, in Rwanda, an AI for health programme supported by the Rwandan government and the African Development Bank, provides training to healthcare professionals in AI-driven diagnostic tools [19]. This initiative enhances AI literacy, enabling local healthcare workers to integrate AI into clinical practice and improve healthcare outcomes, particularly in resource-constrained settings.

We recommend a community-driven approach to deploying AI to ensure robust community engagement and cultural relevance. For example, establishing strategic AI and data science centres of excellence with links to the community in academic institutions could foster innovation and provide a hub for research and development tailored to regional and community health challenges. Building partnerships between such academic institutions and healthcare providers can ensure sustainability. Moreover, involving local health workers and indigenous leaders in co-designing AI tools ensures the solutions align with cultural values and address specific health needs.

## COSTS AND INVESTMENTS

There is a growing body of evidence on the costs associated with deploying AI and data science in the health system [20]. However, these costs remain variable and generally high [7,21], implying careful planning and budgeting when designing and/or implementing AI technologies. The initial setup costs of creating a functional digital health system and climate response centre, which typically involves procuring hardware, software, and other essential technologies, are substantial for many African governments [21], with many seeking financial support from institutions like the World Bank and the International Monetary Fund to support their initiatives [22].

Investments in high-performance computing resources, cloud storage, AI platforms, and skilled information technology professionals, all essential for developing, deploying, and maintaining AI-driven solutions, are estimated to cost the United States between USD 200 billion and USD 360 billion annually [19,20]. As explained by some authors [23], the current level of investment in developing healthcare AI among members of the Global Digital Health Partnership does not seem to yield a high return yet. This is mainly due to underinvestment in the supporting infrastructure necessary to enable the successful implementation of AI.

An innovative financing model is the use of climate (green) bonds, an alternative financing instrument to the classic sources of financing, where governments, financial institutions or corporations raise capital from investors (bonds), which are channelled into climate-related projects [24]. For example, in South Africa, the city of Cape Town issued a green bond in 2017, raising ZAR one billion (approximately USD 75 million) to finance climate-related infrastructure projects. These included initiatives related to water management, renewable energy, and energy efficiency [25]. These bonds could be adapted for funding AI in public health, tying financial returns to measurable improvements in health outcomes.

## CONTEXTUAL CHALLENGES AND THE WAY FORWARD

Asides addressing organisation and costs, deploying data science and AI in African health systems presents several significant challenges, many of which are context-related. Addressing these challenges is crucial for successfully integrating these technologies and maximising potential benefits.

Long-term sustainability is a key issue that can be addressed through local capacity building that ensures African professionals can maintain and update AI systems. As noted, establishing AI research centres in partnership with universities across Africa could support continuous training and adaptation of AI solutions.

Reliable internet and power supply are major challenges in Africa, yet essential for upgrading digital infrastructure and systems. These can be addressed by investing in renewable energy sources like solar and wind power to provide stable electricity, expanding broadband coverage through satellite technology, and partnering with private sector companies to build resilient and affordable digital networks. Yet, despite these measures, the costs need to be well considered.

Ethical and regulatory challenges must be addressed to ensure the responsible deployment of AI in healthcare. There is a need for African governments and policymakers to develop clear regulatory frameworks that govern the use of AI in healthcare, addressing issues such as data privacy, algorithmic bias, and accountability. Governments should establish policies and regulations protecting patient rights while fostering innovation. Implementation of such policies has reportedly been very poor in many settings [26]. Engaging stakeholders, including healthcare providers, patients, and AI experts, in the policymaking process is essential to ensure the regulations are practical and effective.

## CONCLUSIONS

Strategic investments, targeted capacity building, robust regulatory frameworks, and community engagement are needed to successfully deploy and/or integrate data science and AI to address climate-related health risks, like respiratory diseases. We advocate for a targeted phased rollout, starting with AI literacy programmes and piloting AI-driven health monitoring systems in major cities with clear indicators such as improved weather forecasts, reductions in respiratory symptoms or hospitalisations, and improved healthcare access. It is hoped that with continuous investments, adaptation, improvements and scaling of interventions, African nations can report measurable progress and improved population health outcomes following the deployment of data science and AI in public and respiratory health.
